# Novel Treatments for Thymoma and Thymic Carcinoma

**DOI:** 10.3389/fonc.2015.00267

**Published:** 2015-11-30

**Authors:** Arun Rajan, Heather Wakelee, Giuseppe Giaccone

**Affiliations:** ^1^Thoracic and Gastrointestinal Oncology Branch, National Cancer Institute, National Institutes of Health, Bethesda, MD, USA; ^2^Department of Medicine, Division of Medical Oncology, Stanford University, Stanford, CA, USA; ^3^Lombardi Comprehensive Cancer Center, Georgetown University, Washington, DC, USA

**Keywords:** thymic epithelial tumors, autoimmune paraneoplastic disorders, genomic changes, anti-cytokine antibodies, biological therapies

The rarity of thymic epithelial tumors (TETs) creates unique challenges in conducting translational research and developing newer paradigms of treatment (1). Nevertheless, steady progress in genome research, the development of thymic cell lines, and an increase in collaborative efforts between various institutions have helped in fostering an increase in the understanding of the biology of TETs and associated paraneoplastic syndromes and given an impetus to the development of newer treatment options for patients with these rare tumors.

Within the last few years, a number of unique genomic changes have been described in TETs (2). Thymic carcinomas have been found to frequently harbor mutations in epigenetic regulatory genes (3). A specific mutation in GTF2I on chromosome 7 is present at a high frequency in World Health Organization (WHO) subtype A and AB thymomas (4). A nine-gene signature has been developed to predict the metastatic behavior of thymomas (5). The ongoing study of thymoma as part of The Cancer Genome Atlas (TCGA) project promises to add significantly to the existing body of knowledge about genomic alterations in TETs.

The association between thymomas and autoimmune paraneoplastic disorders is well recognized (6). Some patients with thymoma are also more susceptible to infectious complications due to underlying immune dysregulation (7). Various pathogenic mechanisms implicated in the development of immune dysfunction include decreased expression of the autoimmune regulator (AIRE) gene and the presence of anti-cytokine antibodies (8–13). These discoveries have influenced the screening and management paradigms for patients with thymoma.

Treatment strategies for newly diagnosed and recurrent TETs have also evolved over time. Surgery is considered the cornerstone of management of early stage TETs and complete resection of the tumor has a major impact on prognosis (14). For locally advanced disease, thymectomy with en bloc removal of all involved structures is indicated (15). Active areas of investigation focusing on the surgical management of TETs include an evaluation of minimally invasive surgery and an assessment of the role of surgery for recurrent TETs (16–19). Though of unproven benefit and with some controversy, post-operative radiotherapy is recommended after resection of stage III and IVA TETs, and can be considered for stage II thymic carcinoma and cases of stage II thymoma at high risk for recurrence (WHO B3 histology; extensive transcapsular invasion) (15, 20, 21). Chemotherapy is used for induction therapy in cases of stage III/IVA TETs and for treatment of unresectable and recurrent disease (15). There is scant evidence to support the use of chemotherapy after resection of TETs (22).

Contributions to the research topic on “Novel Treatments for Thymoma and Thymic Carcinoma” highlight recent developments in the understanding of the biology of TETs and review various aspects of management of TETs. Huang and colleagues describe previously unreported changes in the expression of apoptosis-related genes in WHO subtype B3 thymomas and thymic squamous cell carcinomas (23). These changes include up-regulation of the anti-apoptotic gene BIRC-3, overexpression of the BIRC-3 protein, and reduced expression of the pro-apoptotic gene, MTCH2 in thymic squamous cell carcinomas, and reduced expression of the pro-apoptotic gene, PMAIP1/NOXA in WHO subtype B3 thymomas. These discoveries have potential therapeutic implications since drugs targeting BIRC-3 and PMAIP-1 are in development (24).

Martinez and Browne review immunological deficiencies associated with thymoma and suggest a paradigm for comprehensive immunological evaluation in patients with thymoma, which should include an assessment of quantitative immunoglobulins, lymphocyte phenotyping, a vaccine challenge in patients suspected to have antibody deficiency and detection of anti-cytokine antibodies, whenever possible (25). Possible therapeutic interventions include immunoglobulin replacement in patients experiencing recurrent sinopulmonary infections due to immunoglobulin deficiency, and use of topical or systemic antifungal drugs in patients susceptible to chronic mucocutaneous candidiasis due to the presence of IL-17 or IL-22 antibodies (25).

Shapiro and Korst discuss the role of surgery for thymic tumors with pleural involvement (26). Surgical approaches that can be considered in this setting include metastasectomy for patients with a limited number of pleural lesions and extrapleural pneumonectomy for patients with more extensive pleural involvement. Data supporting the potential utility of intraoperative, hyperthermic, intrathoracic chemotherapy are also discussed and the need for prospective clinical trials to firmly establish the role of surgery for the management of stage IVA TETs is highlighted.

The role of radiation therapy in the management of thymic epithelial tumors is reviewed by Komaki and Gomez (27). The indications for adjuvant and definitive radiation therapy are discussed as well as techniques to deliver radiation and the long-term effects of mediastinal radiation therapy.

Finally, the review by Chen and colleagues focuses on the latest advances in systemic therapies for TETs (28). Results from clinical trials evaluating novel biological therapies including histone deacetylase inhibitors, insulin-like growth factor inhibitors, and multikinase inhibitors are discussed, and ongoing phase II trials for TETs are highlighted.

The aforementioned manuscripts provide a snapshot of important research efforts related to TETs. Continued advances in the field have resulted in an ever increasing stream of data that offer newer insights into the biology of these rare tumors and support the use of newer paradigms of management for patients with thymoma and thymic carcinoma.

## Author Contributions

Conception and design: all authors; manuscript writing: all authors; final approval of manuscript: all authors.

## Conflict of Interest Statement

The authors declare that the research was conducted in the absence of any commercial or financial relationships that could be construed as a potential conflict of interest.
